# Short-Term Outcomes of Elective High-Risk PCI with Extracorporeal Membrane Oxygenation Support: A Single-Centre Registry

**DOI:** 10.1155/2022/7245384

**Published:** 2022-09-16

**Authors:** Alexander M. Griffioen, Stijn C. H. Van Den Oord, Marleen H. Van Wely, Gerard C. Swart, Herbert B. Van Wetten, Peter W. Danse, Peter Damman, Niels Van Royen, Robert Jan M. Van Geuns

**Affiliations:** ^1^Radboud University Nijmegen Medical Centre, Nijmegen, Netherlands; ^2^Rijnstate Hospital, Arnhem, Netherlands

## Abstract

**Background:**

If surgical revascularization is not feasible, high-risk PCI is a viable option for patients with complex coronary artery disease. Venoarterial extracorporeal membrane oxygenation (VA-ECMO) provides hemodynamic support in patients with a high risk for periprocedural cardiogenic shock.

**Objective:**

This study aims to provide data about short-term outcomes of elective high-risk PCI with ECMO support.

**Methods:**

A retrospective single-center registry was performed on patients with high-risk PCI receiving VA-ECMO support. The short-term outcome was defined as the incidence of major adverse cardiac events (MACE) during the hospital stay and within 60 days after discharge.

**Results:**

Between January 2020 and December 2021, 14 patients underwent high-risk PCI with ECMO support. The mean age was 66.5 (±2.5) and the majority was male (71.4%) with a mean left ventricular ejection fraction of 33% (±3.0). Complexity indexes were high (STS-PROM risk score: 2.9 (IQR 1.5–5.8), SYNTAX score I: 35.5 (±2.0), SYNTAX score II (PCI): 49.8 (±3.2)). Femoral artery ECMO cannulation was performed in 13 patients (92.9%) requiring additional antegrade femoral artery cannula in one patient because of periprocedural limb ischemia. The mean duration of the ECMO run was 151 (±32) minutes. One patient required prolonged ECMO support and was weaned after 2 days. Successful revascularization was achieved in 13 patients (92.8%). Procedural success was achieved in 12 patients (85.7%) due to one unsuccessful revascularization and one procedural death. MACE during hospital stay occurred in 4 patients (28.6%) and within 60 days after discharge in 2 patients (16.7%).

**Conclusion:**

High-risk PCI with hemodynamic support using VA-ECMO is a feasible treatment option, if surgical revascularization is considered very high risk. Larger and prospective studies are awaited to confirm the benefits of ECMO support in elective high-risk PCI comparing ECMO with other mechanical circulatory support devices, including coaxial left cardiac support devices and IABP. *Trial Registration*. This trial is registered with NCT05387902.

## 1. Introduction

Revascularization of complex coronary artery disease including multivessel coronary artery disease, left main stenosis, bifurcation stenosis, and chronic total occlusion (CTO) can be performed by percutaneous coronary intervention (PCI) or coronary artery bypass graft (CABG), according to current guidelines [1]. In order to decide whether to revascularize by either CABG or PCI, patient characteristics, the presence of comorbidities, including diabetes, and coronary lesion complexity (e.g., SYNTAX) should be taken into consideration. Because of the aging population with a higher incidence of comorbidities and higher surgical risk scores, high-risk PCI in complex coronary artery disease with a high risk for periprocedural cardiogenic shock is increasingly performed [2, 3].

Mechanical circulatory support (MCS) devices for elective high-risk PCI can provide hemodynamic support preventing hemodynamic failure during PCI. Several studies are performed using an intraaortic balloon pump (IABP) and coaxial left cardiac support device (Impella device (Abiomed, Danvers, USA)) without showing any clear benefits for IABP. The Impella with its larger hemodynamic support showed some advantages although the official primary endpoint was not reached [4–9]. Venoarterial extracorporeal membrane oxygenation (VA-ECMO) is an alternative option for mechanical support options providing more extensive hemodynamic support in patients with the potential or ongoing failure of circulation. This concept has mainly been demonstrated in postcardiotomy settings or other severe cardiogenic shock patients. ECMO has the additional benefits of right ventricular unloading and blood oxygenation as opposed to IABP and Impella [10]. Our experience with fully percutaneous VA-ECMO in cardiogenic shock and for extracorporeal cardiopulmonary resuscitation (ECPR) combined with the expertise of large bore access management related to transcatheter aortic valve replacement (TAVR) with local anesthesia and mild sedation resulted in the selection of VA-ECMO with local anesthesia as our preferred method in high-risk PCI. Studies investigating the use of VA-ECMO support during high-risk PCI are, however, limited [11–14]. Therefore, the aim of this study is to provide additional data concerning the short-term outcomes of elective high-risk PCI with VA-ECMO support in a single PCI center.

## 2. Methods

### 2.1. Study Population

A single-center, retrospective registry was performed, including all patients who underwent high-risk PCI with VA-ECMO support between January 2020 and December 2021 in the Radboud University Medical Centre (Nijmegen, The Netherlands). Mechanical circulatory support (MCS) was indicated by the HeartTeam based on patient and lesion characteristics described in the expert consensus on the use of MCS devices for high-risk PCI [15]. Patients with the following criteria were eligible: coronary artery disease of the left main, a last remaining conduit or severe multivessel disease, taking the SYNTAX score into account; with a severely impaired left ventricular ejection fraction (LVEF), defined as LVEF ≤35% or decompensated heart failure, defined as the presence of heart failure with clinical symptoms requiring treatment; rejected for CABG as a primary treatment option. Patients who underwent non elective PCI with VA-ECMO support, primarily applied for cardiogenic shock or ECPR, were excluded. The Dutch Act on Medical Research involving Human Subjects (WMO) does not apply to this study, as declared by the ethical committee, because of the retrospective design of the study using only medical records and because no additional study-related procedures are performed. Therefore, no written informed consent was obtained from patients or legal representatives as well.

Patient characteristics, including age, gender, comorbidities, and cardiac status, STS predicted risk of mortality (STS-PROM) risk score, SYNTAX score I and II, and LVEF as assessed by a transthoracic echocardiogram (TTE), nuclear imaging. or magnetic resonance imaging (MRI) were analyzed. Procedural characteristics including target vessels, number of stents, stent characteristics, and revascularization success were collected as well as the duration of ECMO support, ECMO-related complications, and vascular closure technique. Follow-up data, including admission at the intensive care unit (ICU) and the occurrence of major adverse cardiac events (MACE), were collected up to 60 days after PCI.

### 2.2. Definitions and Study Endpoints

Successful revascularization was defined as final residual stenosis <50% with a TIMI flow grade 3, achieved in at least one of the target vessels. Procedural success was defined as angiographic success without the occurrence of periprocedural MACE, including death and myocardial infarction (MI). Additionally, MACE was assessed during the hospital stay and within 60 days follow-up after discharge and defined as a composite of death, MI, target vessel revascularization (TVR) by PCI or CABG, and clinical bleeding, assessed by the Bleeding Academic Research Consortium (BARC) scale. Bleeding complications of type 2 and higher were included. The Valve Academic Research Consortium (VARC)-2 consensus document was used to assess major and minor vascular access sites or access-related complications [16].

### 2.3. Statistical Analysis

Categorical variables are expressed as numbers (with percentages). Continuous data are analyzed for Gaussian distribution and expressed as means (± standard deviations (SD)) or medians (interquartile ranges (IQR)). Baseline patient and angiographic characteristics of patients with VA-ECMO support and ECMO standby are analyzed. Categorical variables are analyzed using the *χ*^2^-test or Fisher's exact test. Normally distributed continuous data are analyzed using an independent *t*-test. Skewed continuous data are analyzed using the Mann-Whitney *U* test. Statistical analysis is performed with IBM SPSS Statistics Software Version 25.0 (IBM Corp., Armonk, NY, USA).

## 3. Results

### 3.1. Baseline Clinical and Angiographic Characteristics

**Figure 1:**
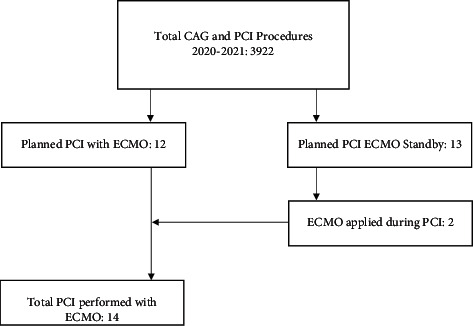
Flowchart of PCI procedures performed with ECMO support. CAG, coronary angiography; ECMO, extracorporeal membrane oxygenation; PCI, percutaneous coronary intervention.

Between January 2020 and December 2021, 3922 patients underwent acute or elective coronary angiography (CAG) or PCI in the Radboud University Medical Centre. Elective PCI with planned VA-ECMO support was the strategy in 12 patients (0.31%). PCI with ECMO standby, implicating that all preparations are made to perform VA-ECMO support in case of cardiogenic shock, was planned in 13 patients (0.33%). In 2 of these patients, step-up to VA-ECMO was implemented. The other 11 procedures were completed with conventional inotropic agents. As a result, a total of 14 patients underwent high-risk PCI with VA-ECMO support (Figure 1).

The mean age of the study population was 66.5 (±2.5). The majority was male (71.4%). Only 3 patients (21.4%) underwent prior PCI. Three-vessel coronary artery disease was present in 57.1% of the study population. At least 11 patients (78.6%) had a left main (LM)-stenosis. In the majority of the population, a chronic total occlusion (CTO) was present (71.4%). The mean left ventricular ejection fraction was 33% (±3.0). The median STS mortality score was 2.9 (IQR 1.5–5.8), the mean SYNTAX score I was 35.5 (±2.0), the mean SYNTAX score II (PCI) was 49.8 (±3.2), and the mean SYNTAX score II (CABG) was 36.2 (±3.1). The indication for mechanical hemodynamic support was a severe multivessel disease or left main coronary artery disease with severely impaired LVEF in the majority of the study population (57.1%). Three patients (21.4%) received hemodynamic support because of severe multivessel disease or left main coronary artery disease with (recent) heart failure. MCS was applied in 3 patients (21.4%) because of extensive coronary artery disease with expected technically challenging and prolonged PCI. One patient was admitted to the ICU before the PCI procedure because of an out-of-hospital cardiac arrest (OHCA). Baseline patient and angiographic characteristics of patients with planned VA-ECMO support and ECMO standby are summarized in Table 1.

### 3.2. Procedural and ECMO Characteristics

A total number of 34 vessels was planned for treatment. The number of successfully treated target vessels with TIMI III flow and residual stenosis <50% was 30 (88%). A total number of 55 stents were implanted with a total stent length per patient of 100 (±16.2) mm. The mean number of drug-eluting stents (DES) implanted for each vessel was 1.6 (±0.2) with a mean stent length of 25.0 (±1.6) mm. The mean procedural time was 202 (±24) minutes and the mean amount of contrast used was 224 (±69) ml. Procedural characteristics are summarized in Table 2.

ECMO cannulation was performed in the common femoral artery in 13 patients (92.9%). One patient required an additional antegrade femoral perfusion cannula because of periprocedural limb ischemia. One patient required cannulation in the axillary artery because of severe peripheral arterial disease resulting in extensive femoral calcifications and stenosis. The median size of the arterial cannula was 17 (17–19) French (Fr). The mean duration of the ECMO run was 151 (±23) minutes. One patient received prolonged ECMO support as immediate weaning failed and was weaned after 2 days. The mean ECMO flow was 2.5 (±0.2) l/min. The majority of the patients received local anesthesia with only preprocedural medical sedation (57.1%). Percutaneous closure was predominantly performed using a suture-based closure device (Perclose ProGlide™ system (Abbott Vascular, CA, USA)) in 9 patients (69.2%) and a collagen plug-based device (Manta, Teleflex, PA, USA) in 3 patients (23.1%). ECMO insertion was performed by surgical cutdown in one patient, and therefore, vascular closure was also performed surgically. In one patient, closure was not performed as a result of periprocedural death. An ECMO-related minor vascular access site complication occurred in one patient with profound bleeding after deployment of the Perclose ProGlide™ system (Abbott Vascular, CA, USA), assessed according to the VARC-2 consensus as percutaneous closure device failure, and was treated successfully with FemoStop™ Femoral Compression System (Abbott Cardiovascular, IL, USA). ECMO characteristics are summarized in Table 3.

### 3.3. Outcomes

Successful revascularization was achieved in 13 patients (92.8%). In one patient, retrograde crossing of the aorticostial occlusion of the left main was unsuccessful. Procedural success was achieved in 12 patients (85.7%), of which 7 patients had improvement of clinical symptoms or LVEF. One patient was not successfully revascularized. For one patient in whom ECMO was initially on standby as a bailout strategy, rupture of one of the PCI target vessels resulted in tamponade and hemodynamic collapse for which ECMO was inserted. Despite ECMO and attempts to revascularize the target vessels, the patient's condition deteriorated, resulting in periprocedural death. Although in one patient with ongoing ECMO support, a perforation of the coronary artery occurred, resulting in bleeding requiring treatment with a covered stent, the patient was immediately successfully weaned from ECMO after achieving antegrade TIMI 3 flow. A total of 4 patients (28.6%) were admitted to the intensive care unit (ICU) after the PCI procedure: one patient for recovery after general anesthesia, one patient for observation, one patient for prolonged VA-ECMO support, and one patient who was already admitted to the ICU ward for optimization of heart failure before the procedure. In-hospital MACE occurred in 4 patients (28.6%), including one patient who died periprocedural, one patient who died 8 days after a PCI procedure because of cardiac failure due to myocardial infarction without any treatment options, one patient requiring target vessel revascularization, and one patient with coronary bleeding, assessed as BARC 2, requiring additional treatment. After hospital discharge, one patient died of end-stage heart failure and one patient died of a myocardial infarction, which could be caused by stent thrombosis. No further MACE occurred within 60 days after discharge (Table 4).

## 4. Discussion

The current study was performed to provide additional data about the short-term outcomes after high-risk PCI with VA-ECMO support. This single-center registry showed that PCI procedures with VA-ECMO support, performed in the majority of the patients with percutaneous access and only local anesthesia, resulted in successful revascularization with limited complications during the hospital stay and in the first 60 days after discharge, despite the high complexity of coronary artery disease.

### 4.1. High-Risk PCI with ECMO Support

It has already been demonstrated that ECMO can be a life-saving treatment for patients with cardiogenic shock [17]. Current guidelines state that mechanic circulatory support may be considered in patients with acute coronary syndrome (ACS) or cardiogenic shock [1]. Additionally, the use of ECMO has been reported to result in beneficial outcomes in patients with out-of-hospital cardiac arrest (OHCA). [18, 19]. However, data on the outcome of mechanical circulatory support in elective high-risk PCI patients are limited, especially with VA-ECMO.

Successful revascularization was achieved in 13 patients (92.8%). Additionally, procedural success was achieved in 12 patients (85.7%). Remarkably, in patients with elective PCI with ECMO support procedural success was achieved in 12 patients (100%).

The one procedural death was a patient selected for standby ECMO. Although the time delay from the onset of circulatory collapse to full circulatory support was limited, LV function did not recover rapidly which might suggest that in retrospect, this strategy was of limited value. Subsequently, weaning from ECMO support and long-term abilities to improve LV function were not attempted because of a failure to cross major branches and a preprocedural agreement to not perform surgical intervention.

During a hospital stay, one patient died 8 days after the PCI procedure because of cardiac failure due to myocardial infarction without any treatment options.

After discharge, MACE occurred in only 2 patients (16.7%). Although the exact cause of death of one patient is unclear, stent thrombosis could be a possible explanation for myocardial infarction. However, it is unclear if this one death is attributable to the PCI procedure or the ECMO support.

Evaluating the baseline and angiographic characteristics of the four patients who died during the procedure or during follow-up, no clear pattern is visible to understand these deaths further which could have resulted in new insights to make different treatment strategies in patients with comparable characteristics in the future. Although LVEF was low, respectively, between 15 and 26%, comparable measurements of LVEF were present in patients with good outcomes. Additionally, complexity indexes, including STS-PROM risk score and SYNTAX score I and II, showed comparable values between the patients with good outcomes and the four patients who died during the procedure or follow-up.

Four patients received general anesthesia (28.6%), of which one patient was already mechanically ventilated and therefore sedated at the ICU. The percutaneous suture-based closure technique was used in the majority of the patients using the Perclose ProGlide™ system (Abbott Vascular, CA, USA) or collagen plug-based closure device (Manta, Teleflex, PA, USA). One patient needed surgical closure because of the initial surgical placement of the ECMO cannulas due to a small and extensive calcified femoral artery. In only one patient, a minor vascular access site complication occurred, assessed as percutaneous closure device failure, and was treated successfully.

These results are in general compared to other recently published studies, with a study population with similar complexity of coronary artery disease [13, 14].

Because of limited evidence, according to expert consensus, high-risk PCI with MCS devices should currently be performed only in patients with specific patient and anatomic characteristics [15, 20]. These characteristics include coronary artery disease of the left main, a last remaining conduit, or severe multivessel disease, especially in patients who are inoperable with a severely impaired LVEF or decompensated heart failure [15]. If surgical revascularization is not an option, decided by the heart team, because of high mortality or procedural risk, PCI with mechanical circulatory support could provide an important alternative for patients with often a poor prognosis. Our study population generally meets the criteria of the expert consensus, showing sufficient revascularization results with limited PCI and ECMO-related complications. This study demonstrates the feasibility and safety of VA-ECMO support in high-risk PCI with promising outcomes. Nevertheless, although results seem promising, the use of VA-ECMO should be investigated in prospective and larger studies.

### 4.2. Comparison with Mechanical Circulatory Support Devices

Although the use of VA-ECMO for hemodynamic support for high-risk PCI is not investigated thoroughly, several studies have already been published about the use of other mechanical circulatory support devices, including the Impella and IABP. Perera et al. [4] showed that the use of IABP did not reduce the incidence of MACE after PCI in patients with complex coronary artery disease and severe left ventricular ejection fraction, although long-term follow-up showed a decrease in all-cause mortality in patients treated with hemodynamic support [5]. The safety and feasibility of the Impella were demonstrated by Henriques et al. [6], reporting no procedural and device-related deaths. Additionally, several registries are performed to assess the outcomes of high-risk PCI with Impella support as well. These registries showed some positive results regarding the survival rate and the incidence of MACE [7–9]. Despite the relatively high prevalence of complications, Chieffo et al. [9] noticed that the use of the Impella device is increasing. The PROTECT II Trial [21] compared IABP with Impella in patients with severely impaired LVEF and complex three-vessel coronary artery disease, last remaining vessel, or left main coronary artery disease. This study randomly assigned 452 patients to IABP or Impella, achieving satisfying angiographic results in almost all patients in both treatment groups. Results showed no differences in the 30-day incidence of MACE (per-protocol analysis: 34.3% for Impella versus 42.2% for IABP, *p*=0.092), although 90-day incidence showed a benefit to Impella. In the per-protocol analysis, a decreased incidence of MACE was observed for Impella compared to IABP (40.0% vs. 51.0%, *p*=0.023). In addition, especially in patients with extensive coronary artery disease, the use of Impella results in beneficial outcomes in comparison to IABP (relative risk (RR): 0.78, 95% CI: 0.61–0.99, *p*=0.039).

Each MCS device has particular characteristics. ECMO characteristics differ from IABP and Impella, regarding the ability of hemodynamic support, oxygenation [10], and left ventricle (LV) unloading [22]. Compared to other mechanical devices, ECMO increases LV afterload, therefore theoretically increasing the risk of decompensated heart failure [22]. Russo et al. [23] showed the potential benefit of LV unloading with additional mechanical devices, including IABP, in patients on ECMO support resulting in lower mortality. Additionally, the use of VA-ECMO was previously limited due to large bore access (17 or 19 French vs. 14 French for Impella) with surgical cutdown and general anesthesia. With the extending experience in percutaneous large bore access and dedicated closure devices, local anesthesia with mild sedation is realistic in the majority of patients with mobilization similar to transcatheter aortic valve replacement (TAVR) patients. Because guidelines are currently based on expert consensus, future studies are awaited comparing outcomes between ECMO and other mechanical support devices, such as IABP and the Impella, in patients with high-risk PCI.

### 4.3. Limitations

This study has some limitations. First, it is limited by the retrospective design, resulting in an increased risk of missing data. Second, the population is of limited size. Additionally, selection bias might have occurred because of the highly selected population. Third, because this study does not include a control group, no conclusions can be made about any preferential treatment.

## 5. Conclusion

High-risk PCI with hemodynamic support using VA-ECMO is a feasible treatment option if surgical revascularization is considered as very high risk. Larger and prospective studies are awaited to confirm the benefits of ECMO support in elective high-risk PCI, comparing ECMO with other mechanical circulatory support devices, including coaxial left cardiac support devices and IABP.

## Figures and Tables

**Table 1:** Baseline patient and angiographic characteristics.

	PCI with ECMO support (*n* = 14)	PCI with ECMO standby (*n* = 11)	*P*value
Age, years (±SD)	66.5 (±2.5)	69.8 (±2.5)	0.37
Male, % (*n*)	71.4 (10/14)	72.7 (8/11)	1.00
Hypertension, % (*n*)	42.9 (6/14)	45.5 (5/11)	1.00
Diabetes mellitus, % (*n*)	57.1 (8/14)	45.5 (5/11)	0.56
Dyslipidaemia, % (*n*)	28.6 (4/14)	36.4 (4/11)	1.00
Congestive heart failure, % (*n*)	57.1 (8/14)	27.3 (3/11)	0.23
Prior MI, % (*n*)	57.1 (8/14)	63.6 (7/11)	1.00
Prior PCI, % (*n*)	21.4 (3/14)	36.3 (4/11)	0.67
Peripheral artery disease, % (*n*)	28.6 (4/14)	9.1 (1/11)	0.34
Lung disease, % (*n*)	14.3 (2/14)	27.3 (3/11)	0.62
Chronic kidney disease (GFR <30 ml/min), % (*n*)	7.1 (1/14)	18.2 (2/11)	0.57
Dialysis	100 (1/1)	100 (2/2)	
Three-vessel coronary artery disease, % (*n*)	57.1 (8/14)	54.5 (6/11)	1.00
Left main stenosis, % (*n*)	78.6 (11/14)	81.1 (9/11)	1.00
Generic bifurcation lesion, % (*n*)	21.4 (3/14)	36.4 (4/11)	0.67
CTO, % (*n*)	71.4 (10/14)	63.6 (7/11)	1.00
LVEF, % (±SD)	33 (±3.0)	36 (±4.6)	0.54
STS mortality risk score (± SD or IQR)	2.9 (1.5–5.8)	2.7 (±0.5)	0.55
SYNTAX score I (±SD)	35.5 (±2.0)	32.0 (±2.3)	0.28
SYNTAX score II (PCI) (±SD)	49.8 (±3.2)	51.5 (±3.7)	0.74
SYNTAX score II (CABG) (±SD)	36.2 (±3.1)	40.1 (±2.8)	0.38
Indication for MCS, % (*n*)			
LM, SRC, or SMVD with severe LV-dysfunction	57.1 (8/14)		
LM, SRC, or SMVD with (recent) HF	21.4 (3/14)		
LM, SRC, or SMVD with expected technically challenging and prolonged PCI	21.4 (3/14)		
Preprocedural admission at ICU, % (*n*)	7.1 (1/14)	0 (0/11)	1.00

CTO, chronic total occlusion; ECMO, extracorporeal membrane oxygenation; GFR, glomerular filtration rate; HF, heart failure; IQR, interquartile range; LM, left main; LVEF, left ventricular ejection fraction; MCS, mechanical circulatory support; MI, myocardial infarction; N, number of treated patients; PCI, percutaneous coronary intervention; SD, standard deviation; SMVD, severe multivessel disease; SRC, single remaining conduit.

**Table 2:** Procedural characteristics: PCI target vessels (*n* = 34).

Vessels planned for treatment, % (*n*)	
LM, % (*n*)	32 (11/34)
LAD, % (*n*)	29 (10/34)
LCx, % (*n*)	18 (6/34)
RCA, % (*n*)	21 (7/34)
Number of vessels planned for treatment per patient (±SD)	2.4 (±0.3)
Number of treated vessels, % (*n*)	88 (30/34)
Number of treated vessels per patient (±SD)	2.1 (±0.3)
Total number of stents implanted	55
Total stent length per patient, mm (±SD)	100 (±16.2)
Number of stents (±SD)	1.6 (±0.2)
Mean stent length, mm (±SD)	25.0 (±1.6)
Mean stent diameter, mm (±SD)	3.2 (±0.1)
Procedural time, min (±SD)	202 (±24)
The contrast used, ml (±SD)	224 (±69)

ECMO, extracorporeal membrane oxygenation; LAD, left anterior descending; LCx, left circumflex; LM, left main; N, number of treated vessels; PCI, percutaneous coronary intervention; RCA, right coronary artery; SD, standard deviation.

**Table 3:** ECMO characteristics: PCI with ECMO support (*n* = 14).

ECMO cannulation, % (*n*)	
Femoral artery	92.9 (13/14)
Femoral antegrade cannula necessary	7.7 (1/13)
Subclavian artery	7.1 (1/14)

Arterial cannula size, Fr (IQR)	17 (17–19)
17 French, % (*n*)	66.7 (8/12)
19 French % (*n*)	16.7 (2/12)
21 French % (*n*)	16.7 (2/12)

Anaesthesia, % (*n*)	
Local anesthesia with preprocedural medical sedation	57.1 (8/14)
Procedural sedation and analgesia	14.3 (2/14)
General anesthesia	28.6 (4/14)

Duration of ECMO, min (±SD)	151 (±23)
Prolonged ECMO support, % (*n*)	7.1 (1/14)
ECMO flow, l/min (±SD)	2.5 (±0.2)

Closure technique, % (*n*)	
Collagen plug-based	23.1 (3/13)
Suture-based closure device	69.2 (9/13)
Surgical	7.7 (1/13)

ECMO-related complications, % (*n*)	
Vascular access site or access-related	7.1 (1/14)
Major vascular complication	0 (0/1)
Minor vascular complication	100 (1/1)

ECMO, extracorporeal membrane oxygenation; Fr, French; N, number of treated patients; PCI, percutaneous coronary intervention; SD, standard deviation.

**Table 4:** Short-term outcomes: PCI with ECMO support (*n* = 14).

Successful revascularization, % (*n*)	92.8 (13/14)
Procedural success, % (*n*)	85.7 (12/14)
Improvement of symptoms or LVEF	58.3 (7/12)
In-hospital MACE, % (*n*)	28.6 (4/14)
Death, % (*n*)	14.3 (2/14)
MI, % (*n*)	6.7 (1/14)
TVR, % (*n*)	6.7 (1/14)
Clinical bleeding, % (*n*)	14.3 (2/14)
BARC type 2	50.0 (1/2)
BARC type 3a	0 (0/2)
BARC type 3b	0 (0/2)
BARC type 3c	0 (0/2)
BARC type 4	0 (0/2)
BARC type 5a	50.0 (1/2)
BARC type 5b	0 (0/2)
MACE within 60 days after discharge, % (*n*)	16.7 (2/12)
Death, % (*n*)	16.7 (2/12)
MI, % (*n*)	8.3 (1/12)
TVR, % (*n*)	0 (0/12)
Post-PCI admission to ICU, % (*n*)	28.6 (4/14)

ECMO, extracorporeal membrane oxygenation; ICU, intensive care unit; LVEF, left ventricular ejection fraction; MACE, major adverse cardiac events; MI, myocardial infarction; N, number of treated patients; PCI, percutaneous coronary intervention; TVR, target vessel revascularization.

## Data Availability

The data used to support the findings of this study are available from the corresponding author upon request.
